# Using Deep Learning and Low-Cost RGB and Thermal Cameras to Detect Pedestrians in Aerial Images Captured by Multirotor UAV

**DOI:** 10.3390/s18072244

**Published:** 2018-07-12

**Authors:** Diulhio Candido de Oliveira, Marco Aurelio Wehrmeister

**Affiliations:** Computing Systems Engineering Laboratory (LESC), Federal University of Technology—Parana (UTFPR), Curitiba 80230-901, Brazil; wehrmeister@utfpr.edu.br

**Keywords:** pedestrian detection, aerial images, Unmanned Aerial Vehicle (UAV), thermal camera, deep learning, convolutional neural network, pattern recognition system, performance assessment

## Abstract

The use of Unmanned Aerial Vehicles (UAV) has been increasing over the last few years in many sorts of applications due mainly to the decreasing cost of this technology. One can see the use of the UAV in several civilian applications such as surveillance and search and rescue. Automatic detection of pedestrians in aerial images is a challenging task. The computing vision system must deal with many sources of variability in the aerial images captured with the UAV, e.g., low-resolution images of pedestrians, images captured at distinct angles due to the degrees of freedom that a UAV can move, the camera platform possibly experiencing some instability while the UAV flies, among others. In this work, we created and evaluated different implementations of Pattern Recognition Systems (PRS) aiming at the automatic detection of pedestrians in aerial images captured with multirotor UAV. The main goal is to assess the feasibility and suitability of distinct PRS implementations running on top of low-cost computing platforms, e.g., single-board computers such as the Raspberry Pi or regular laptops without a GPU. For that, we used four machine learning techniques in the feature extraction and classification steps, namely Haar cascade, LBP cascade, HOG + SVM and Convolutional Neural Networks (CNN). In order to improve the system performance (especially the processing time) and also to decrease the rate of false alarms, we applied the Saliency Map (SM) and Thermal Image Processing (TIP) within the segmentation and detection steps of the PRS. The classification results show the CNN to be the best technique with 99.7% accuracy, followed by HOG + SVM with 92.3%. In situations of partial occlusion, the CNN showed 71.1% sensitivity, which can be considered a good result in comparison with the current state-of-the-art, since part of the original image data is missing. As demonstrated in the experiments, by combining TIP with CNN, the PRS can process more than two frames per second (fps), whereas the PRS that combines TIP with HOG + SVM was able to process 100 fps. It is important to mention that our experiments show that a trade-off analysis must be performed during the design of a pedestrian detection PRS. The faster implementations lead to a decrease in the PRS accuracy. For instance, by using HOG + SVM with TIP, the PRS presented the best performance results, but the obtained accuracy was 35 percentage points lower than the CNN. The obtained results indicate that the best detection technique (i.e., the CNN) requires more computational resources to decrease the PRS computation time. Therefore, this work shows and discusses the pros/cons of each technique and trade-off situations, and hence, one can use such an analysis to improve and tailor the design of a PRS to detect pedestrians in aerial images.

## 1. Introduction

The Unmanned Aerial Vehicle (UAV) is an aircraft that flies without an onboard pilot while it is controlled remotely by a person or a computer system. The UAV is being applied in many sorts of different application fields. The cost reduction and technological advances are some of the reasons for this popularization. Applications in fields such as robotics and computer vision are the most popular. UAV have many benefits compared to land vehicles [1], such as: (i) a UAV has more degrees of freedom to dodge obstacles; and (ii) a UAV can cover a wide area in fewer time [2]. In comparison with computer vision applications using satellites, the UAV shows a remarkable advantage related to the increased amount of details provided in the captured images, allowing the detection of small objects; this is called tactical coverage [3].

The tactical coverage allows using UAV in military and civilian applications, either in life-threatening or tiresome/stressful situations. The life-threatening situations include all situations that may lead to injuries of search and rescue staff, for instance, rescue missions in a hostile environment or in an environment contaminated by some toxicity. The tiresome/stressful situations include tasks such as surveillance in border regions or search and rescue missions in wide areas. All these tasks demand a higher degree of concentration for many hours in a row, which can be difficult for a person to maintain. Many academic studies have been conducted using UAV platforms in several applications: (i) detection and surveillance [1,4,5,6,7,8,9,10]; (ii) agriculture [11,12,13]; (iii) 3D modeling [14]; and (iv) remote sensing [15,16,17].

Object detection is one of the main computer vision applications that employs UAV. This task includes search and rescue missions in areas, the access to which is difficult, wide searching areas or areas affected by natural disasters [18]. Examples of these situations include the nuclear plant accident in Fukushima (Japan), Europe’s borders control, the hurricane disasters in the South of the United States or the disaster caused by Samarco’s dam collapse in Brazil. In all of these situations, surveillance actions are required as soon as possible in order to provide medical assistance effectively to the affected population. Fast and planned actions are crucial for saving as many lives as possible. However, in most of the cases, wide areas must be covered within a minimal amount of time, and thus, many UAV must be employed. As a result, an exploding number of images is captured, and they need to be analyzed in a reduced time frame. This analysis cannot be performed manually by a group of persons since this task is very tiresome, stressful and error-prone. Thus, the autonomous detection of pedestrians in aerial images must be applied.

Computer vision applications using images from UAV are complex because they involve many variables. These applications are sensitive to variations of the captured images: (i) images’ instability; (ii) small objects present a fewer number of pixels; and (iii) changes in camera position and angle due to the degrees of freedom of the UAV [1]. The image instability occurs due to the platform movement and instability, which generates blurred images. UAV position (and its corresponding movement) changes the visual shape of the object, i.e., the target object size and position within the image changes. Such visual changes transform the appearance of the object, hindering the detection task. This behavior is called multi-viewpoint [19]. The multi-viewpoint is influenced by the roll, pitch and yaw movements, i.e., the rotation of the UAV on the *x*-, *y*-, *z*-axes. Image variations strongly influence the image recognition performed by computer vision systems. Visual variations affect the object shape, and thus, the systems are not able to generalize the problem [20] (the term “problem” is used in this text to refer to both: (a) a thing that is difficult to achieve or accomplish; and (b) the process of detecting target objects within a PRS). This leads to a situation in which an autonomous system is efficient in several situations, but not in all possible situations. Such a system cannot be used in applications that require precision, such as surveillance tasks.

Recent studies have concentrated efforts on pedestrian and vehicle detection. We can separate the techniques used into two main approaches: (i) monolithic models [1,7] and (ii) part-based models [5,21]. The monolithic model considers the target object as a single object. On the other hand, the part-based models split the object into several parts. Each part can be learned by a classifier, turning the problem into multiple classification tasks. Most of the recent studies are based on hand-crafted features extracted from the images and submitted to a classifier [1,5,7,21]. However, this traditional pattern recognition approach is not efficient in problems that present a high influence of multi-viewpoint. As an alternative, the deep learning approaches have been applied. The Convolutional Neural Network (CNN) is one of the most successful deep learning techniques employed to classify images [22]. For problems with strong multi-viewpoint influence, the CNN provides efficient solutions, since it is invariant to shift, scale and distortion [23].

Despite the CNN recognition and detection capacity, such a technique requires many computational resources, e.g., processing units and memory. Another traditional technique used to detect pedestrians in aerial images is the sliding window. The algorithm splits an image into parts with a size of M × N pixels creating a sequence of Regions Of Interest (ROI), i.e., the algorithm extracts the first ROI from the pixels at the (0, 0) position, the second ROI from the pixels at (0 + stepX, 0) position, and so on. Thereafter, the classification algorithm is executed on all extracted ROI. Thus, depending on the size of both the image and the window, this technique processes thousands of ROI.

The CNN requires the use of at least one Graphics Processing Unit (GPU) to process the image within soft real-time constraints [22,24]. A GPU is an expensive processing unit (in comparison with regular CPUs) that consumes a higher amount of energy, which may raise several issues for the embedded computing system with which the UAV is equipped. In this situation, the use of a Mobile Ground Control Station (MGCS) emerges as an alternative to process the images captured by the UAV. The captured images are transmitted from the UAV to the MGCS, which, in turn, may use a GPU to process the aerial images. However, such a system architecture demands a reliable communication infrastructure that must fulfill not only the minimal bandwidth requirements but also the time constraints.

MGCS and embedded systems equipped with a GPU are still expensive computing platforms. Their use in conjunction with multiple UAV may create an unaffordable technology for search and rescue forces, especially in less developed regions. More economical computing platforms could help to overcome the cost issues. Therefore, it is worth investigating whether it is possible to decrease the PRS processing time, opening room for using less expensive computing systems in pedestrian detection systems. In this case, techniques to reduce the number of ROI to be analyzed by the classifiers improve the search and detection task performance. These techniques employ simple and efficient algorithms to detect the target objects of interest with a higher match probability, allowing the PRS to respond within a shorter time frame.

In this work, we propose to develop and assess some PRS using distinct machine learning techniques to detect pedestrians in UAV aerial imagery, considering the multi-viewpoint problem and a monolithic model. For that, we used robust techniques for pedestrian detection in images: Haar Cascade, Cascade with Local Binary Patterns (LBP), Histograms of Oriented Gradient (HOG) with Support Vector Machine (SVM) and Convolutional Neural Network (CNN). Considering the CNN, two distinct implementations have been created: one based on the architecture proposed by [22] and the other based on [25]. Both implementations use the Caffe Framework [26]. To reach soft real-time detection constraints, we use two techniques to reduce the number of analyzed ROI: Saliency Map (SM) and Thermal Images Processing (TIP). It is important to highlight that the main contribution of this work is the evaluation and comparison of various PRS implemented using a combination of distinct techniques commonly applied in computer vision systems for pedestrian detection. Although we performed some modifications of the techniques used to implement these PRS, tailoring them to the pedestrian detection application is not a major contribution. We are interested in assessing the entire PRS and some of its steps individually. We aim to identify which combination of techniques can provide feasible and suitable results considering a set of low-cost sensors and processing units, e.g., inexpensive single-board embedded computers (such as Raspberry PI) and laptops without a GPU.

We conducted several experiments to assess the classification performance and the real-time processing capabilities of the developed PRS. For that, we evaluated each technique on its capacity to detect and classify pedestrians within the aerial images in two situations: (a) a person appears entirely in the images; and (b) only a part of the person appears in the images due to partial occlusion. The obtained results show that the CNN has the higher capacity for generalization, achieving 99.71% accuracy, followed by HOG + SVM with 92.36%. In the occlusion scenario, the better result of CNN is 71.1% accuracy. For the computing time performance evaluation, the experiments have been carried out on different platforms without GPU: MGCS (Intel i5-based laptop) and an embedded platform (Raspberry Pi 2). By using CNN and TIP, the PRS achieved a computing time performance of 2.43 fps running on the MGCS and 0.18 fps running on the embedded platform, both reaching a good classification performance (88.15% sensitivity and 99.43% specificity). Combining CNN with SM achieved the best classification performance (92.37% sensitivity and 88.93% specificity), but the computing time performance was 2× worse than CNN + TIP (1.35 fps running on the MGCS and 0.09 fps running on the embedded platform). On the other hand, by using HOG + SVM and thermal image processing, the PRS achieved 100 and 14 fps running on the MGCS and the embedded systems, respectively, both reaching a limited classification performance (62.96% sensitivity and 87.54% specificity). Combining SM, HOG, and SVM provides a better classification performance (74.05% sensitivity and 79.87% specificity), but the computing time performance is worse: 3.33 and 0.25 fps running on the MGCS and the embedded system, respectively. These results show that it is feasible to design a PRS for detecting pedestrians in aerial images that execute on a less expensive computing platform (in this case, the MGCS) with an acceptable computing time performance and a good classification rate.

In summary, this work’s contribution concentrates on the analysis and the assessment of combining distinct computer vision techniques to implement a pattern recognition system that executes on low-cost and resourceless computing systems. Therefore, this work presents the following contributions:

- We evaluated the AlexNet [22] performance (in terms of both classification and computational) on a classification task, which was not the original target application. We performed small modifications on AlexNet in order to allow detecting pedestrians in aerial images.
- We evaluated another CNN architecture [25], which has been successfully used in real-world situations for human biometric identification; not pedestrian detection in aerial images. This CNN presents a fewer number of parameters and layers. Such a feature impacts the computational performance by reducing the number of operations, which in turn, may allow the use of low-cost hardware.
- We created a large dataset of aerial images depicting pedestrians that is suitable for deep learning techniques. For that, we combined existing datasets and performed a data augmentation process.
- We assessed the performance of the classifiers in situations of pedestrian partial occlusion. We did not include any image of an occluded pedestrian in the training dataset and process.
- We performed several experiments in real-world situations. These experiments included low-quality images captured by low-cost cameras affected by the instability generated during the UAV flight. These images have not been used in the training process either.
- We evaluated the classification performance of some traditional computer vision techniques and also their computational performance running on low-cost hardware, e.g., Raspberry PI 2 and a laptop computer that uses an x86-based CPU without GPU. The goal was to check the feasibility and suitability of applying such techniques to low-cost embedded systems, in order to decrease the cost of a UAV equipped with a pedestrian detection system.

The remainder of this paper is organized as follows. Section 2 discusses some related works. Section 3 provides an overview of the important concepts and techniques used in image pattern matching problems. Section 4 details the proposed approach. Section 5 discusses the obtained results. Finally, Section 6 draws some conclusions and indicates some future work directions.

## 2. Related Work

Several methods and techniques have been used in object detection in aerial imagery. Most of these works target the following objects of interest: people, vehicles, forests, towns, and plantations. The methods used can be separated into two main categories: Movement-Based Detection (MBD) and Stationary-Based Detection (SBD). The main difference between these methods is how the regions of interest are pre-classified. However, both methods use machine learning techniques to classify these regions.

The MBD techniques are able to detect objects that present movement between a set of frames [27,28]. This is a serious limitation for search and rescue missions since static objects are ignored and processed as background. On the other hand, SBD uses information from a single frame to detect ROI. This method requires brute-force approaches for segmentation and detection, such as sliding windows, or more sophisticated algorithms to reduce the space of search. An extensively-used technique is thermal image processing [1,10,21,29]. This kind of technology provides thermal information that can be used in applications where the object of interest has a constant temperature, e.g., people or other species of mammals.

In the region classification step, two main methods are used [5]: monolithic models and part-based models. The monolithic model processes the object as a single element; thus, the classification is based on all parts of the object. On the other hand, in the part-based model, the object is decomposed into several elements that can be independent or interconnected. The monolithic model is simplistic, but it has disadvantages in situations of object occlusion. In another way, the part-based model is more robust, but it demands complex machine learning techniques and also requires much information to be processed. For aerial imagery applications, the monolithic model is more suitable, since the objects of interest in these applications have a small set of pixels to be processed.

The study conducted in [5] focused on people detection in indoor environments for search and rescue missions. In fact, the images used by the authors suggested an application closer to pedestrian detection. However, the main contribution of the work was in the situations of object occlusion. Part-based models are used for this task since, in several situations, people are partially occluded by desks, chairs and other objects commonly found in corporate environments. Three algorithms are used in combination: Pictorial Structures (PS) [5], Discriminately-Trained Models (DPM) [30] and Poselet-Based Detection (PBD) [31]. The PS divides the people body image into several independent elements, where the recognition is realized using AdaBoost [32]. Similar to PS, the DPM also splits the person body image into several parts, but in this algorithm, one element is defined as the main element to which all other elements are attached. The DPM uses support vector machine [33] to detect the body elements. The PBD is similar to PS; the difference is that the PBD classifier is executed by the template matching algorithm.

The people detection in aerial imagery is the main subject of [34]. In this work, the rotation on the *x*-, *y*-, *z*-axes (i.e., pitch, roll and yaw) is considered. Using Integral Channel Features (ICF) [35] and Cluster Boosting Tree (CBT) [36], some detectors were proposed. According to the authors, these features and classifiers were chosen due to the good computational performance in situations in which the cameras’ angles change frequently. Three detectors were proposed based on the rotational degrees of freedom: Pitch-trained Detector (PD), Roll-trained Detector (RD) and Pitch- and Roll-trained Detector (PRD). The experiments evaluated the classification rate and processing time and with roll and pitch variation. In comparison with [34], this work employs different feature descriptors and classifiers, as well as it analyzes the model’s performance in some distinct angle ranges. In addition, our work performs an extensive and more comprehensive analysis of the computing time performance.

Some studies [7,37,38] applied shadow detection algorithms to detect people and vehicles in aerial imagery. Shadows are often hindering in this sort of applications because it is difficult to separate the shadow of the object of interest. The work presented in [7] uses shadow detection and also geographic metadata to estimate the shadow position and reduce the time of the classification step. To detect the person in the estimated region, the sliding window algorithm was used; each ROI was processed by a pre-trained SVM with Haar features.

A popular approach used by several authors in the literature [1,4,10,29,39,40] is thermal image processing. Some approaches use only thermal images, ignoring RGB images, while others use thermal images only in the detection process. By using thermal images, it is possible to obtain solutions to the traditional problems in people detection and recognition: low contrast, crowds, changes in visual conditions and others. Thermal images are especially suitable for applications in which the target object temperature is constant. The segmentation process using thermal images does not demand much processing time, making such a technique suitable to be used for embedded applications, such as a UAV equipped with an embedded PRS. In addition, in [4], thermal images were used to detect ROI that have a high chance of depicting pedestrians in aerial images before the classification step, which in turn, is executed on the RGB image. On the other hand, Ref. [1] applied Haar cascade [41] to detect vehicles and people in thermal images, whereas the work presented in [10] applied HOG features and SVM to detect and track people in aerial images in several scenes.

The study conducted in [8] used the image processing algorithm, the Felzenszwalb graph cut method, to detect regions’ disparities and to classify them as a pedestrian or not. Multi-Scale Histogram of Oriented Gradients (MS-HOG) was used in combination with several classifiers. The study reached good results with more than 95% of the ROC curve area, despite the low resolution of the images in the experiments. It is important to notice that PRS implemented in [8] demands a higher processing time, due to a large number of objects detected by the segmentation algorithm. Our work, on the other hand, provides a computing time performance assessment in order to check what combinations of computer vision techniques can execute on low-cost computing devices and also reach the system time constraints.

In general, the studies presented in the literature focused just on recognition or detection process. Several approaches in the literature are not suitable for real-world applications, due to the long processing time of the systems. Furthermore, some detection techniques are not robust enough to deal with several camera angle variations or object pose diversity. In this work, we used a deep learning technique to detect pedestrians in aerial imagery and compared the results with traditional hand-crafted feature approaches that are usually used in this sort of PRS. We also compared the computing time of an entire PRS executing on top of two different computing platforms; the worst-case scenario is analyzed and discussed. Another contribution is the use of low-resolution thermal cameras for detection (80 × 60 pixels resolution), while the mentioned related works used cameras with higher resolution, e.g., at least 320 × 240 pixels.

## 3. Background

### 3.1. Multi-Viewpoint Problem

**Figure 1:**
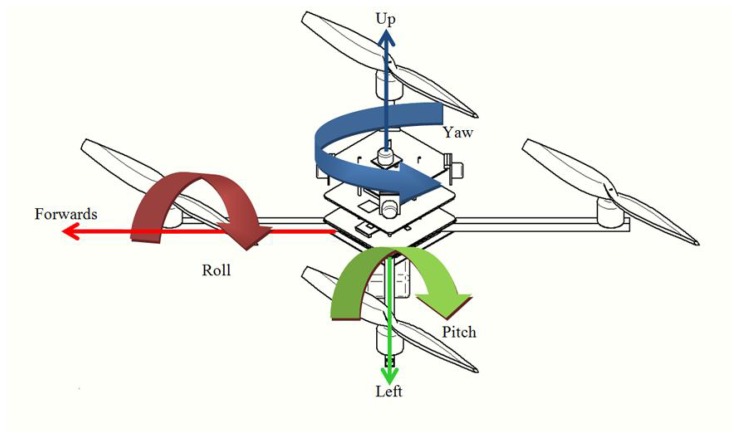
The rotational degrees of freedom of a UAV: roll (*x*-axis), pitch (*y*-axis) and yaw (*z*-axis) [42].

The multi-viewpoint problem is related to computer vision systems running on platforms with several rotational degrees of freedom. In those applications, the platform has the freedom to move itself or the camera (or the entire system) to different directions on one or more axes. UAV platforms are able to move on three axes (*x*, *y* and *z*), generating three different angles: roll (*x*-axis), pitch (*y*-axis) and yaw (*z*-axis). Figure 1 shows the UAV degrees of freedom and rotational angles.

The variation of those rotational angles causes different effects on the objects in the scene, e.g., the roll variation changes the object rotation, the pitch may change the object shape, whereas yaw may modify the object translation [42]. For most machine vision algorithms, the visual changes caused by roll and pitch strongly influence the recognition process since they modify the object appearance, while the yaw changes only the object positioning. It is worth noticing that some algorithms are strongly affected by translation. Thus, when those variations are combined, the object visual variation is much more evident.

Therefore, robust classification methods (i.e., those with high generalization capability) are necessary. These methods require a large-scale dataset that covers all situations of angle variations. In general, the usual pedestrian dataset does not provide a wide variety of viewpoints, only the horizontal one. Thus, for UAV applications, it is necessary to use images collected by the UAV platform. Some image datasets such as Generalized Multi-View Real Training dataset (GMVRT-v1, GMVRT-v2) [34] and UFC-ARG dataset [43] provide images with rotational angle variations.

### 3.2. Pattern Recognition System

Pattern recognition is a field of computer science that has the objective to identify and classify objects in classes using their patterns. This process can be executed with different kinds of data, such as images, wave signals, statistical data and others. The applications are present in several fields of study, such as medicine, psychology, computer science, agriculture, etc.

**Figure 2:**
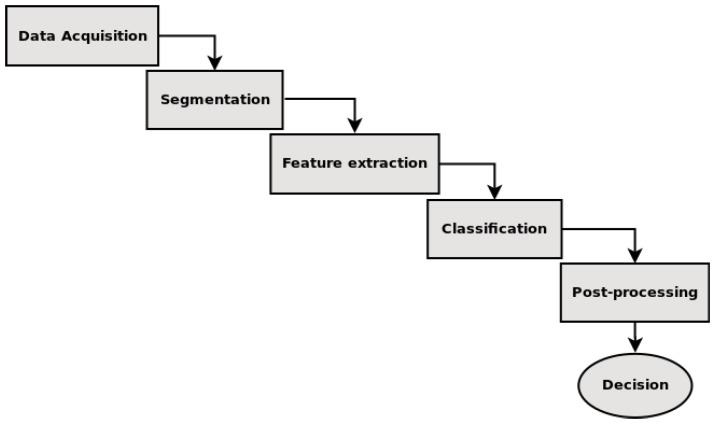
A basic pattern recognition system.

The pattern recognition task must cope with several issues, making this a very complex task. According to [44], a PRS is generally split into five steps: acquisition, segmentation, feature extraction, classification, and post-processing. Figure 2 shows these PRS steps.

The acquisition step refers to extraction of the data submitted to the system. In acquisition, a sensor or specialized hardware gathers a set of data, e.g., radio signals, RGB images, thermal images or audio waves. The segmentation step analyses the input data, looking for objects of interest, i.e., the objects of interest are isolated from the rest of the data. It is important to highlight that objects of interest vary according to the application such as pedestrians in search and rescue applications or potential threads in a surveillance system. The output of this step is a set of Regions Of Interest (ROI). The system uses the segmented ROI to perform the feature extraction. These features are used to make connections among candidates and, thus, define the classes’ patterns. This step is critical because the selected features must reveal representational patterns. Thereafter, in the classification step, the classifier uses the defined features to discover patterns on the input data, dividing the objects into the classes using prior knowledge (usually obtained in a training phase). Lastly, the post-processing step takes some action based on the classification results, producing the system’s final decision. In the post-processing, the objects can be highlighted with a bounding box or the system can send a notification to the search and rescue team warning about new events or detections.

Most of the PRS follow these basics steps, but this is not a strict division of the workflow. Several systems may omit some steps, whereas several others merge two or more steps. The deep learning techniques are examples of merging steps in which feature extraction and classification are executed within the same algorithm [45]. Furthermore, the system flow varies in some applications, especially in those that need a feedback of information from the previous steps [44]. The feedback is used when more than one classifier is used; a classifier can use the data processed by a previous one.

In order to reach a desirable performance, it is important to select a representative set of features and establish the appropriate classifiers. The classification step requires typical samples of the application domain. These samples must be segmented into and as into classes, in order to acquire prior knowledge about the target problem and its classes. These samples must include the maximum number of samples for each class, including variations. The group of samples is named for the dataset. A representative dataset allows the classifiers to generalize the problem, which in turn, improves the results in real-world applications [44]. After the training phase, the classifier works as a model [46]. Given an input sample, the respective object class must be outputted by the classifier. The training process is named supervised training if the training samples are labeled; otherwise, it is named unsupervised training [44]. In the unsupervised training, the classifier splits the dataset into an arbitrary number of classes following the samples’ similarity, according to pre-defined criteria.

As mentioned, this work aims at evaluating different implementations of PRS that combine distinct computer vision techniques and algorithms. Each PRS follows the basic structure as discussed above. For acquisition, we used RGB and thermal images obtained from low-cost cameras. In the segmentation step, we used Saliency Map (SM) and Thermal Image Processing (TIP). The feature extraction and classification steps are performed in the following way. We combined Haar and Local Binary Patterns (LBP) with the cascade AdaBoost classifier (i.e., two combinations: Haar + cascade and LBP + cascade) and also Histograms of Oriented Gradient (HOG) with the Support Vector Machine (SVM) classifier. On the other hand, when the deep learning techniques were used, we merged feature extraction and classification, which were implemented within two distinct architectures of Convolutional Neural Networks (CNN). Section 4.5 discusses these combinations of techniques. Details of these PRS implementations and their assessment are provided in the following sections.

## 4. Materials and Methods

### 4.1. Overview of the Research Method

**Figure 3:**
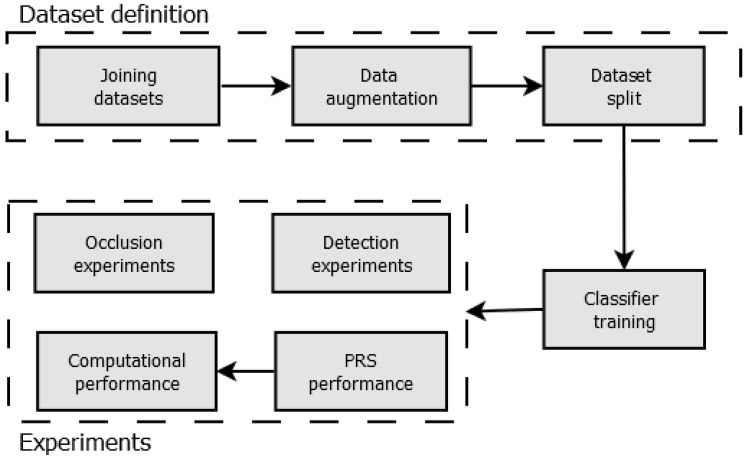
Overview of the research method.

This work is focused on autonomous pedestrian detection in UAV aerial imagery for search and rescue missions. For that, we used the pattern recognition systems concepts. Therefore, it is necessary to establish an adequate classifier and to train it properly. Following the PRS concept, we defined a workflow based on a dataset that contains several situations in pedestrian detection. These situations include pedestrians in different poses, as well as distinct rotational degrees of the aerial platform. Thus, we used the dataset to train, validate and test the classifiers. Lastly, we performed some experiments using the proposed techniques in several real-world situations in order to evaluate the combined techniques in terms of both classification capacity and computational performance. Figure 3 shows the workflow used in this work.

In the first step, we defined a consistent dataset for pedestrian detection in aerial imagery. The dataset contains the objects of interest in several situations, i.e., people in several activities. Moreover, the dataset contains images captured by onboard cameras of aerial platforms including several variations of roll, pitch and yaw angles. Since the CNN needs a large-scale set of data for the training step [47], we conducted a data augmentation process to increase the number of samples in the dataset.

Following the traditional training procedure of machine learning algorithms, it is necessary to split the dataset into training, validation and test sets [44]. The training samples are used to train the classifier and to tune its parameters. The validation samples are used in the classifier training to evaluate the training result and improve the parameters’ adjustment. The validation process is used to prevent the overfitting, i.e., when the classifier becomes too closely or exactly adjusted to the training data. An overfitted classifier is not able to classify new samples submitted to the classifier correctly; only the ones used during training. We executed the classifier training according to the best parameters (see more details in Section 4.4) adjusted for our image datasets. We used the same set of images for all classifiers for the training, validation, and test. Thereafter, the test images are used to validate the classifier model created in the training step. Finally, it is worth mentioning that, by combining distinct computer vision techniques, we implemented and assessed five distinct PRS. We combined Haar features and LBP features with the cascade classifier, which are extensively used in the literature [29,48,49]. We combined HOG features with the SVM classifier since it is widely used in pedestrian detection [50,51]. We used the convolutional neural networks due to the solid results obtained in object identification competitions [22] and other applications for people detection [24,25].

In the experiment phase, we carried out four sorts of experiments: (i) detecting objects of interest (detection experiments); (ii) detecting objects of interest that are partially occluded; (iii) PRS classification performance and (iv) PRS computing time performance executing on two distinct hardware platforms. In the detection experiments, we assessed Haar + cascade, LBP + cascade, HOG + SVM and two architectures of CNN on their capacity to classify pedestrians in aerial imagery correctly. The occlusion experiments are aimed at assessing the classifiers’ capability of generalization. For that, we added the image of a tree overlapping the pedestrian image artificially. This procedure hides a portion of the object of interest simulating the partial occlusion, which is a common situation in aerial imagery.

Finally, we evaluated the PRS detection and computational performance. For that, we varied the implementation of the PRS by combining the five classifications methods with two distinct segmentation techniques: Saliency Map (SM) and Thermal Image Process (TIP). Therefore, ten distinct combinations were implemented and assessed in terms of classification and computing time performance. The classification performance was evaluated by means of accuracy, sensitivity and specificity metrics. The computing time performance was evaluated by measuring the execution time of the ten PRS combinations on two low-cost computing platforms: an inexpensive single-board embedded computer (Raspberry PI 2) and a regular laptop without a GPU. Details are provided in the following sections.

### 4.2. Images Dataset

In this study, we merged some image datasets to create a larger dataset that presents pedestrians in several poses. The dataset includes a wide diversity of images in several rotational angles captured by onboard cameras on UAV platforms. We selected three image datasets: GMVRT-v1 [42], GMVRT-v2 [34] and UCF-ARG Dataset [43]. These datasets contain aerial RGB images of pedestrians (positive samples) and non-pedestrians (negative samples). All samples are segmented images. Most of these images depict pedestrian silhouettes. It is important to notice that these RGB images were used in the classifiers’ training and testing, whereas the thermal images were used just in PRS experiments.

**Figure 4:**
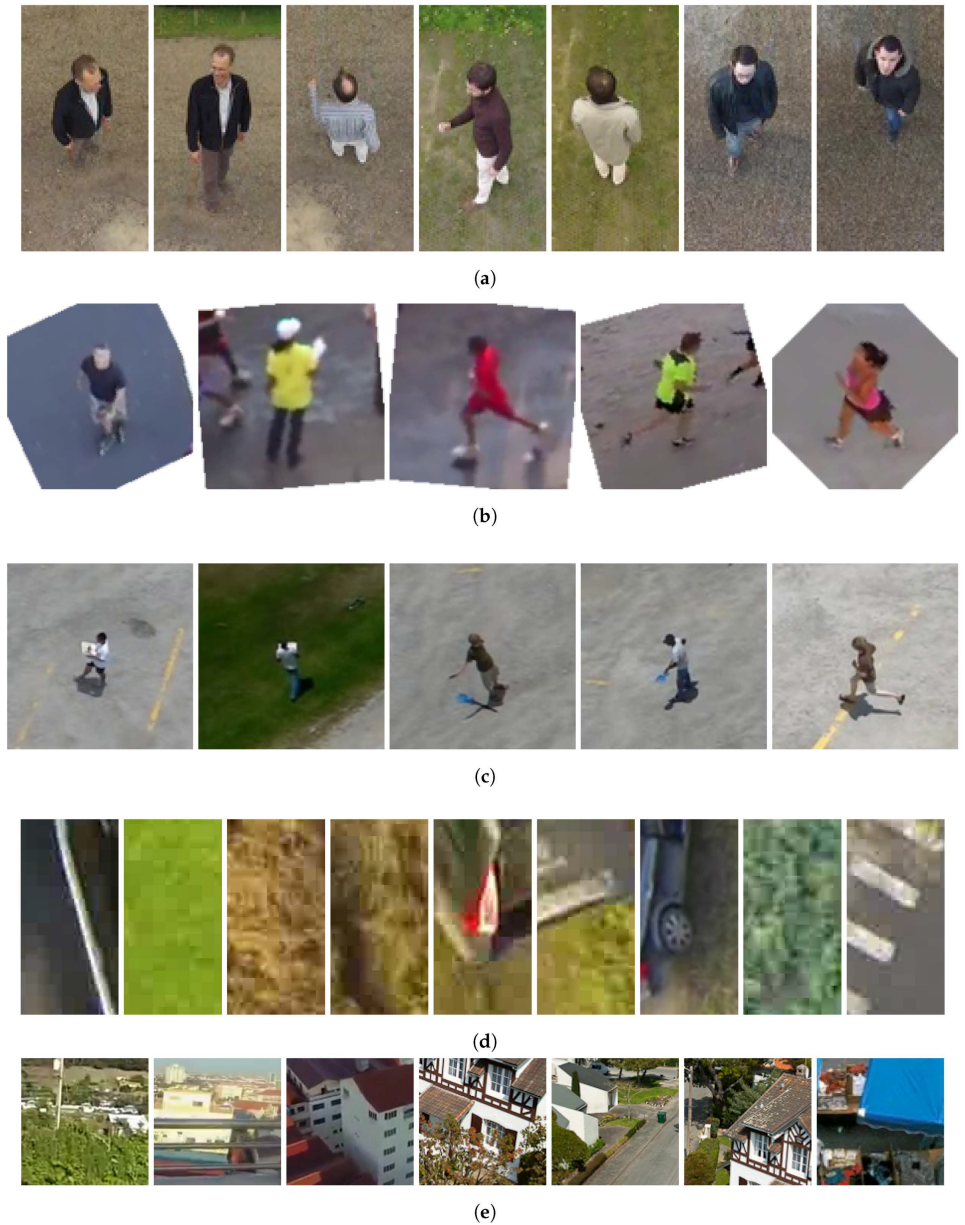
Samples of the aerial images datasets used in this study: (**a**) pedestrian images from GMVRT-v1; (**b**) pedestrian images from GMVRT-v2; (**c**) pedestrian images from UCF-ARG; (**d**) non-pedestrian images from GMVRT-v1 and (**e**) non-pedestrian images from GMVRT-v2.

The image dataset GMVRT-v1 [42] contains pedestrian and non-pedestrian images. It presents a broad variety of pedestrian images in several rotational angles, roll, pitch, and yaw, with a variation between 0${}^{\circ }$ and 90${}^{\circ }$. The dataset provides images of pedestrians in several poses with different kinds of clothes. The negative samples (non-pedestrian) are a set of urban and countryside segments of objects. Originally, the images were available in the RGB format with a color depth of 24 bits per pixel in JPEG format and a resolution of 64 × 128 (width × height) pixels. The dataset contains 4223 positive images and 8461 negative samples. Figure 4a,d depicts some samples of the GMVRT-v1 dataset.

The GMVRT-v2 dataset [34] provides 3846 pedestrians samples in a large variety of poses and rotational angles. While the GMVRT-v1 contains images of several people in the same environment, the GMVRT-v2 contains pedestrians on beaches, streets, grass, sidewalks and other places. Moreover, the dataset provides 13,821 negative samples with the same variety of places. The pedestrian images are segmented into 128 × 128 pixels, 24 bits and PNG format. In addition to the training samples, the GMVRT-v2 contains a set of 210 test images. These images contain three or four pedestrians in the rural environment. The camera angles vary between 45${}^{\circ }$ and 90${}^{\circ }$. To increase the number of samples to train and test the classifiers, we selected two positive samples of each image with a size of 128 × 128 pixels. A total of 420 new pedestrian samples in PNG format has been added to the dataset. The original images of the GMVRT-v2 test set are in JPEG format with a size of 1280 × 720 and 24 bits of color depth. Figure 4b,e shows some samples of the GMVRT-v2 dataset.

The UCF-ARG dataset [43] is a video dataset. It provides a total of 1440 videos that have been captured from three cameras from different point views: aerial, terrestrial and on a rooftop. For each camera position, the dataset contains 480 videos with a resolution of 960 × 540 pixels and 30 fps. These videos show pedestrians executing ten sorts of actions, e.g., punching, carrying objects, walking and digging. We extracted one frame randomly from the 480 videos from the aerial perspective. From these frames, we selected 410 frames and extracted manually the pedestrian images with 128 × 128 pixel resolution in PNG format. A total of 70 frames were excluded because the pedestrian images presented noise or distortions Such a dataset is an interesting dataset for pedestrian applications since it provides images with small variations of scale and poses for the same pedestrian. Figure 4c depicts some samples of the UCF-ARG dataset.

**Figure 5:**
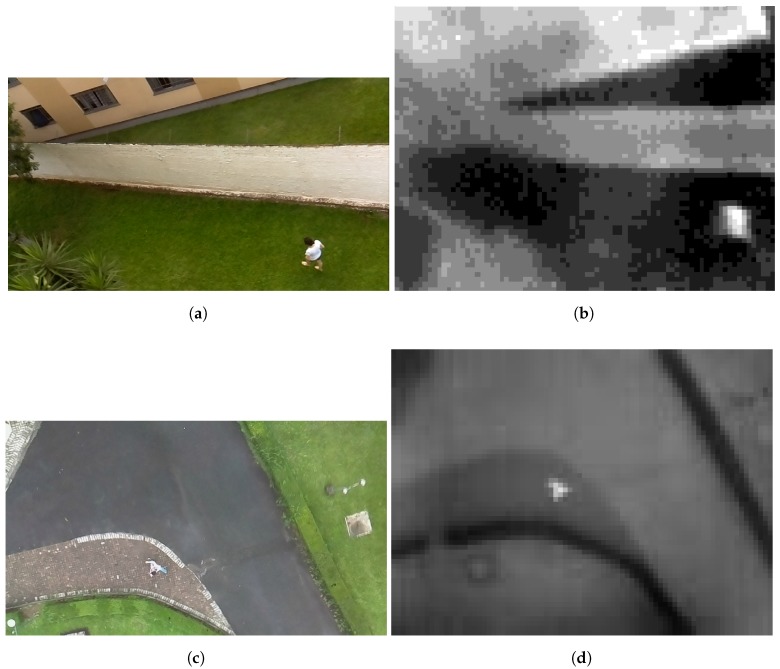
Images captured during the PRS experiments. Subfigures (**a**,**c**) are RGB images captured by the Raspicam; subfigures (**b**,**d**) are thermal images captured by the FLIR camera. We collected the images (**a**,**b**) on the top of a building, whereas we collected (**c**,**d**) during a quad-rotor UAV flight.

**Figure 6:**
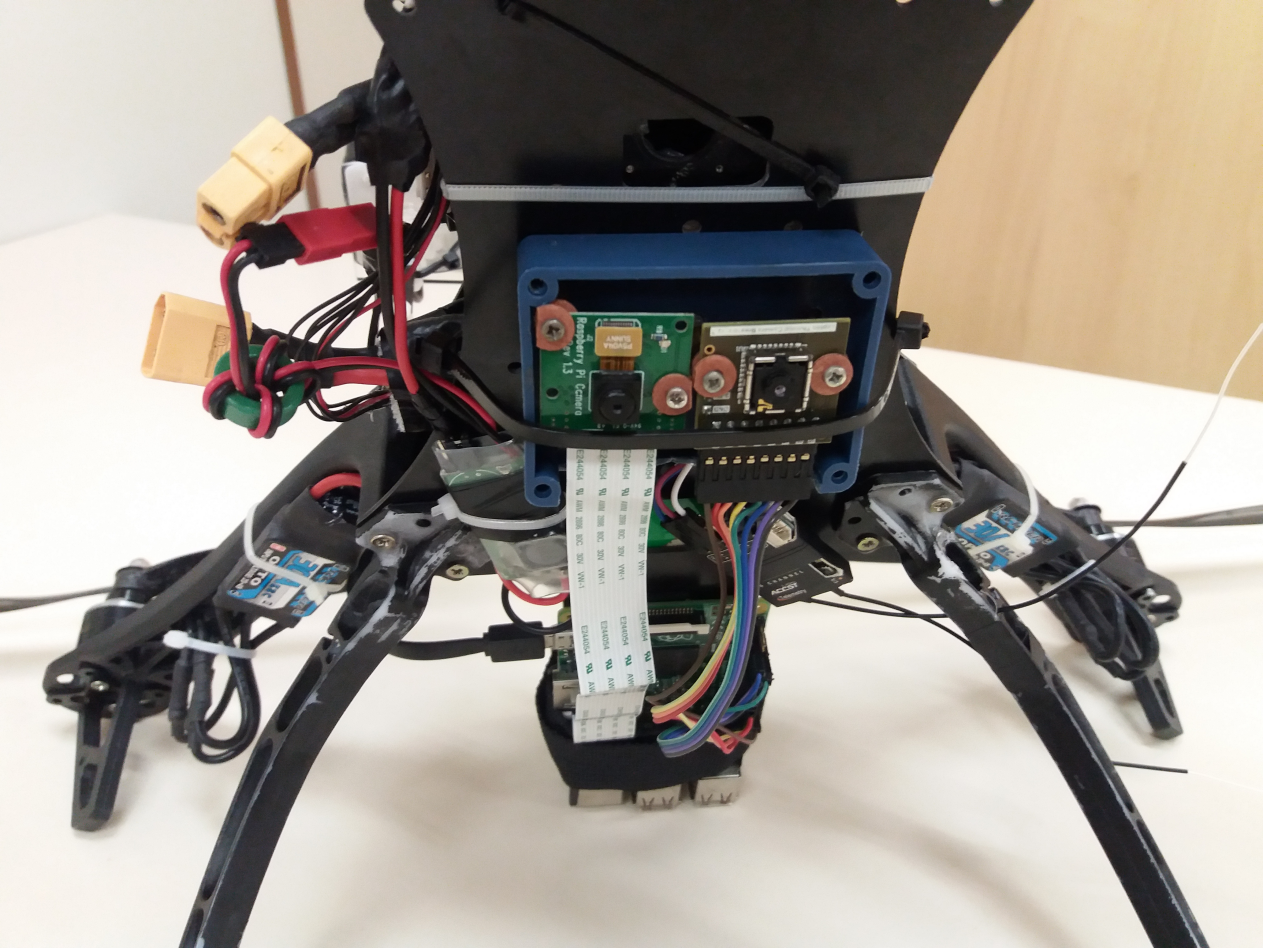
Quad-rotor platform used to collect images for PRS experiments.

For the PRS experiments, we also collected a set of seventeen RGB and thermal images. The RGB and thermal cameras were positioned on the top of a building to simulate the situation of UAV flying with an altitude of 20 m. The target environment is a garden between two buildings. We obtained the RGB images with a resolution of 1280 × 720 pixels and thermal images with 80 × 60 pixels, both with 24-bit color depth and PNG format. Figure 5 shows the RGB and thermal images used in the experiments. We also collected images using a quad-rotor UAV to perform an experiment similar to the real-world situation. Figure 6 shows the UAV platform used in the image extraction. The platform comprises one FLIR Lepton Long Wave Infrared sensor, one Raspicam and one Raspberry Pi 2. The videos were collected in three environments: (i) a grass field; (ii) a grass field with a building and (iii) a street with the sidewalk. For that, we collected eight RGB and eight thermal short videos with the quad-rotor UAV flying at an altitude between 15 and 20 m. Each video has a length of 30 s and 10 fps. Each RGB video was collected with a resolution of 1280 × 720, whereas the thermal video has a resolution of 80 × 60. We extracted randomly 15 frames from each video to perform the PRS experiments. Actually, we selected only 15 frames from each video since we performed the analysis manually. It is important to highlight that these images contain several situations commonly found in the real world such as blurred objects and images with noise. Moreover, each image depicts only one person, and hence, in the 120 selected images, 120 people must be identified. Finally, we merged the images captured in these two ways (i.e., by flying with the UAV and also with the cameras positioned on top of a building) into a single dataset. Therefore, this image dataset contains 137 images with 137 people in real-world situations. Such a dataset can also be considered a contribution of this work since it is available for others to use (see https://github.com/lesc-utfpr/pedestrian-detection).

### 4.3. The Dataset Augmentation

The convolutional neural network is a type of feed-forward artificial neural network. CNN is a machine learning method that uses an extensive set of parameters during the training process. However, like other traditional machine learning techniques, the CNN is susceptible to the curse of dimensionality [52], i.e., as the problem dimensionality increases, more data are necessary to build a model that satisfies the problem requirements and criteria. As the CNN is larger than an ordinary neural network, the number of weights to balance the network increases, leading to the curse of dimensionality problem. For most of the computer vision applications, it is very hard to acquire a large image dataset. An enormous effort is also necessary to label the data for a supervised training process. An option to decrease the effect of the curse of dimensionality is to perform the data augmentation process, i.e., artificially augmenting the number of samples in a dataset. The image dataset augmentation consists of generating “new” samples applying small modifications and also filters on the original dataset samples. In addition to increasing the amount of data, the data augmentation is useful to balance classes when the dataset is unbalanced.

**Figure 7:**
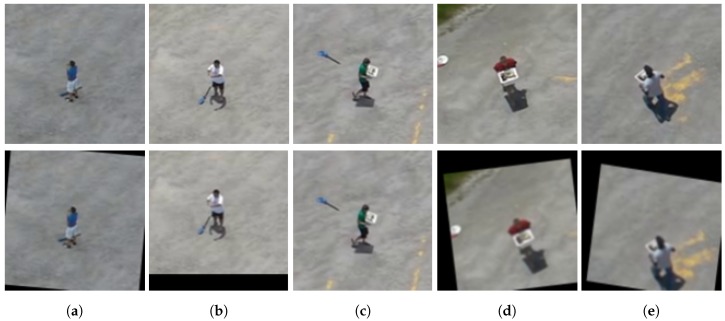
Data augmentation process: original and new images generated by applying the following transformations: (**a**) rotation, (**b**) translation; (**c**) scale; (**d**) rotation and translation and (**e**) rotation, translation and scale.

In this work, we applied a data augmentation process similar to [25]. The process consists of image processing filters that randomly change the original dataset samples, and thus, each sample can be modified by one or more image transformation filters. The transformations performed randomly are: (i) translation (±20 pixels on *x* and/or *y* axis); (ii) scale (between 0.95 and 1.2) and/or (iii) rotation (between −20${}^{\circ }$ and 20${}^{\circ }$). Each transformation is performed on a given sample with a probability of 50%. Figure 7 shows some new samples created during the data augmentation process.

After the data augmentation, the number of dataset images increased from 31,184 samples to 44,765, i.e., an increase of 13,381 positive sample images. The augmentation process created a balance in that the dataset provides 22,483 pedestrian samples and 22,282 non-pedestrian samples.

### 4.4. Classifiers Training

In this work, we applied different classifiers to train and detect pedestrians in aerial imagery: the cascade classifier, SVM, and CNN. These classifiers have a distinct training process and parameter definitions. In order to achieve better results, we executed some preliminary training with all classifiers, and according to the results, we defined the most suitable parameters for each classifier. For this process, we split the dataset into three parts: training, validation and test samples. We split the dataset using the holdout cross-validation [44]. We intended to use k-fold cross-validation. However, due to time constraints for using the GPU cluster to train the neural networks, we chose to use holdout (instead of k-fold) to have more time to run more experiments. The dataset was split following a given proportion. Each sample set is used once in the process, i.e., the training set is used during training to train the classifier, the validation set is to validate the classifier while it is being trained, whereas the test set is used to test the trained classifier. A sample defined as a training sample was not used in the validation or test. This process ensures that the classifier will not be affected by overfitting, allowing the model to generalize the problem efficiently. In this work, we split the dataset as follows: 80% of the samples for training, 10% for validation and 10% for the test.

We executed the cascade training using the implementation available in the OpenCV library. This implementation does not require validation; thus we discard the validation set sample. We trained the classifiers using Haar and LBP features descriptors with the classic AdaBoost version [53]. Cascade was configured as follows: 15 levels in training, false alarm rate of 0.5 and true positive rate of 0.95.

Similar to the cascade classifier training, we trained the SVM using the OpenCV implementation without executing a validation step. The SVM was trained with HOG features, a linear kernel and stop criteria of $1\times 10^{-6}$ error or 100 thousand iterations. We defined the HOG features with nine bins, a block size of 32 × 32 and a cell size of 16 × 16, which totaled 8100 features per sample.

Unlike cascade and SVM, the CNN requires many more parameters for configuration. The CNN requires the architecture definition: a set of convolutional filters must be defined to automatically extract representational features. In this work, we used two CNN architectures, which were named CNN1 and CNN2.

**Figure 8:**
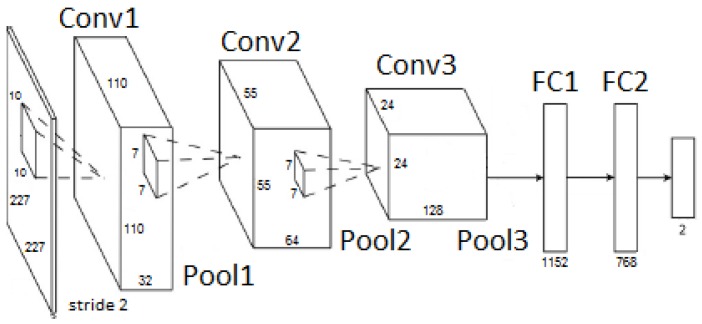
CNN1 architecture.

The CNN1 architecture was proposed in [25] for human biometric identification based on clothes and gender. The CNN1 achieved high accuracy results (greater than 70%). We used the CNN1 architecture because it is used in an application related to people identification, and hence, it has a good chance to achieve good results in pedestrian identification in aerial imagery. Moreover, this architecture was defined with a lower number of layers that may reduce computing time processing since a reduced number of floating point and dot product operations need to be executed. It is important to mention that the computational performance of this CNN architecture was not assessed in [25]. Figure 8 and Table 1 show the CNN1 architecture. The first six layers (Conv1, Pool1, Conv2, Pool2, Conv3 and Pool3) are related to the feature extraction. These feature extractors are defined during the training step, according to the training sample information. As one can see, there is a ReLU max function between Convolutional (Conv) and Pooling (Pool). The ReLU (rectifier) function is the activation function. ReLU max allows non-linearity instead of linear activation functions commonly used in CNN. The following two layers are called fully-connected layers, and they are responsible for the classification using the features extracted in the previous layers. Fully-connected layers are similar to shallow neural networks (i.e., the conventional neural networks), in which all neurons are connected to each other. The last layer produces the final decision, i.e., the classification result. As the problem has two classes, this layer comprises two neurons; one to represent the pedestrian classification and the other to indicate the non-pedestrian classification.

The CNN2 architecture is the well-known AlexNet that was proposed in [22]. This architecture is widely used in various CNN applications since it was the first CNN used in the ImageNet Large Scale Image Recognition Competition (ILSVRC) [54]. In ILSVRC 2012, AlexNet beat the second-place competitor by 9.7% accuracy. Recently, other effective architectures [55,56] have been proposed and used in the ILSVRC; it is worth mentioning that the top rates in this competition have been obtained by CNN approaches. We chose to use the AlexNet architecture due to its reduced number of layers and low complexity in comparison with other CNN architectures and also because we did not have access to a dataset with millions of samples. As we already commented, the larger the number of CNN layers, the larger the dataset needed to obtain adequate results. Moreover, the pedestrian detection in UAV imagery is an application that presents soft real-time constraints, and thus, a complex CNN can hardly meet such constraints running on a low-cost computing system such as an embedded system.

**Figure 9:**
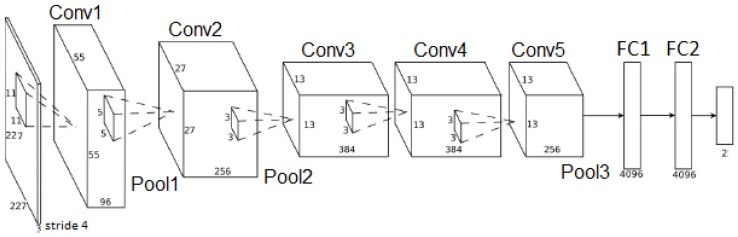
CNN2 architecture.

Figure 9 and Table 2 show the AlexNet architecture. The first eight layers (Conv1, Pool1, Conv2, Pool2, Conv3, Conv4, Conv5 e Pool3) are dedicated to feature extraction. A ReLU function acts as the activation function after each convolutional layer. Similar to CNN1, CNN2 contains two fully-connected layers to execute the classification step, and also, the last layer presents two neurons that correspond to pedestrian and non-pedestrian classes. It is important to highlight that the original AlexNet was adopted in this work. As the target application presents only two classes (i.e., pedestrian and non-pedestrian), we reduced the number of output neurons from 1000 to two neurons in the last CNN layer.

For training both CNN architectures, we used the training and validation sets with holdout cross-validation. The weights are adjusted by the back-propagation algorithm with SoftMax as the loss validation metric [23]. We used the Step Gradient Descent (SGD) algorithm to optimize the CNN. We adjusted the learning rate to 0.005, which is increased by the product of 0.1 every 30 epochs. Furthermore, we used 0.9 as the momentum used to converge the training method. Lastly, we defined the maximum number of epochs as 1000. We defined the number of epochs based on our preliminary experiments [4] in which the CNNs converged between 600 and 800 epochs. We defined the 1000 epochs limit to prevent any loss if any CNN took more epochs to converge. To avoid overfitting, we applied the dropout technique with a 50% rate. The dropout randomly excludes some neurons of the fully-connected layers on each training step with a probability of 50%. This technique is widely used in CNN and significantly improves the network generalization process. These parameters have been used in [22,25].

The CNN training process demands an enormous amount of processing due to a large number of parameters and weights that need to be adjusted during the process. Therefore, we executed the training using a general purpose GPU. The GPU is able to provide a faster training by applying the mini batch approach [22]. The mini batch selects a set of images that are loaded into the GPU memory; these images are used in the weights’ adjustment with back-propagation on each step. According to [22], the mini batch influences the loss function; the smaller the mini batch, the more instability the loss function presents. Thus, a large mini batch results in stability, but it requires a GPU with more memory. In this work, we used a mini batch of 128 images for each step.

### 4.5. Combining Computer Vision Techniques to Implement PRS for Pedestrian Detection in Aerial Imagery

**Figure 10:**
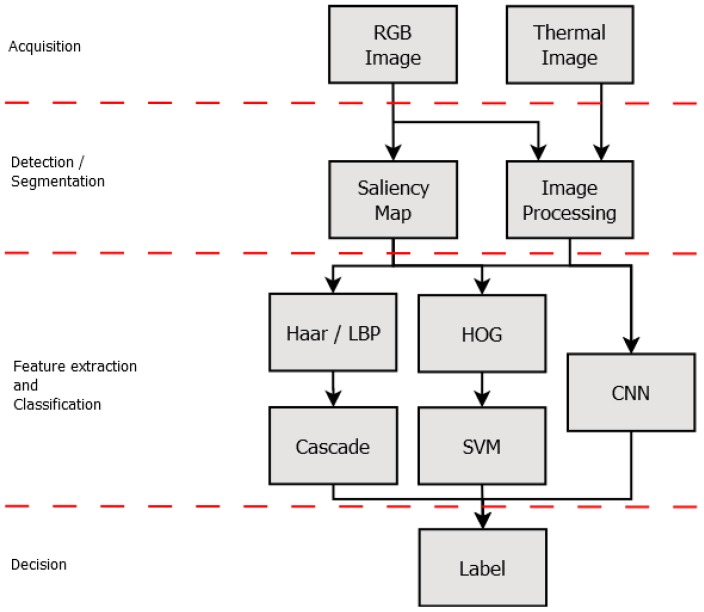
The pattern recognition systems used in this work.

As mentioned, one of the goals is to evaluate the feasibility and suitability of combining distinct computer vision techniques to implement a PRS to detect pedestrians in aerial images captured by onboard cameras of multi-rotor UAV. Therefore, we implemented 10 different PRS. Each PRS was evaluated on its classification capacity in two situations: (i) when the pedestrians appear entirely in the images and (ii) when some part of the pedestrians is hidden due to partial occlusion. Furthermore, these PRS are expected to run on top of low-cost computing platforms, e.g., single-board embedded computers such as the Raspberry Pi or regular laptops without a GPU. Therefore, we performed: (i) an assessment of classification performance; and (ii) an assessment of the computational time performance. The set of implementations follows the PRS steps discussed in Section 3.2. Figure 10 shows the PRS steps and the techniques used in each step. As one can see, we combined two segmentation methods with five features and classifiers, resulting in 10 implementations of pedestrian detection PRS.

In the acquisition step, we used two low-cost sensors: a thermal camera and an RGB camera. The former has a FLIR Lepton Long Wave Infrared sensor (http://cvs.flir.com/lepton-data-brief) that captures thermal images with a resolution of 80 × 60 pixels (the maximum resolution of the device) at 60 fps. The FLIR camera seems a fair alternative to be used in this sort of application, instead of an expensive solution using thermal cameras with higher resolutions, which may cost much more than the UAV itself. The latter is a Raspicam camera module v1 (https://www.raspberrypi.org/documentation/hardware/camera/README.md). Raspicam captures images with a maximum resolution of 2592 × 1944 up to 15 fps; they are sent via a serial interface. To keep the coherence between the images generated by the thermal and RGB cameras, the acquisition processes were executed in parallel. Hence, the images generated by both cameras contain information of the same scene captured almost at the same time instant. It is important to mention that a difference of 10 ms may exist between one thermal image and one RGB image due to the difference in the frame rate of the cameras. However, this difference is not significant in the detection and classification process for the target application.

In the segmentation step, we used two methods to segment and detect the pedestrian: Saliency Map (SM) and Thermal Image Processing (TIP). SM uses the RGB images to detect small discrepancies in the scene. The SM algorithm takes the RGB image as input and outputs a grayscale image with small discrepancies. In our PRS, the grayscale image is submitted to a thresholding process that transforms the image into a binary image. Such a binary image allows a faster object search process since the algorithm needs only to look for pixels the value of which is equal to 1. Thus, the Regions Of Interest (ROI) are extracted when the algorithm finds a set of neighbor pixels the value of which is 1. This ROI is checked before being submitted to the feature extraction. Objects that show a width or height less than 256 pixels are discarded, to remove regions that hardly contain an ROI that depicts pedestrians. Objects with higher dimensions are normalized; thus, all objects submitted to the classifiers have the same dimensions of the training samples: 256 × 256 pixels.

On the other hand, the TIP segmentation detects pedestrians by checking their thermal signature. The thermal image was captured in grayscale format and was submitted to a thresholding process, as well. Pixels whose value is between 95 and 105 (the gray values’ interval between 95 and 105 corresponds to temperatures between 29 ${}^{\circ }$C and 36 ${}^{\circ }$C, approximately) receive a value of 1, whereas the other pixels receive a value of 0. We defined this interval during the experimentation by observing the thermal images and comparing them with the corresponding RGB image. The detection process is similar to the SM process, i.e., the ROI is identified as a set of neighbor pixels the value of which is 1. In the TIP segmentation, the thermal image ROI are not submitted to the classifier. The ROI is extracted from the corresponding RGB image by using the centroid of the thermal image ROI as the reference. The following equations are used to identify the centroid of the RGB image ROI based on the thermal image ROI:

(1) $$x^{\prime }=\frac{W_{rgb}}{W_{thermal}}x$$

(2) $$y^{\prime }=\frac{H_{rgb}}{H_{thermal}}y$$

where *x* and *y* are the centroid coordinates in the thermal image ROI, *W* is the image width and *H* is the image height. By using the calculated centroids, an ROI with a size of 256 × 256 pixels is extracted from the RGB image and submitted to the classifier.

After the segmentation and detection, the ROI was submitted to the trained classifiers. Since we used deep learning techniques to extract features and classify them, we consider these two steps as a single one. Therefore, the following techniques were combined: (i) Haar features and cascade; (ii) LBP features and cascade; (iii) HOG features and SVM; (iv) CNN1; and (v) CNN2. Using the parameters and the model constructed during the training phase, each ROI extracted from the image samples were submitted to all of these classifiers, which, in turn, classify the ROI as either pedestrian or non-pedestrian.

Finally, in the decision step, the image objects were labeled according to the result provided by the classification step. In order to better visualize the results, the ROI classified as a pedestrian are highlighted with red rectangles in the image.

## 5. Experiments and Results

### 5.1. Experiment 1: Detection Experiments

#### 5.1.1. Overview

First of all, we executed some experiments following the Holdout cross-validation to analyze the classifiers’ robustness and generalization capability and also to ensure this did not converge to overfitting. After the classifiers’ training, we evaluated the classifiers’ models using the test set. For applications such as detecting pedestrians in aerial imagery, the classification produces two outcomes: the evaluated sample is either pedestrian (positive) or non-pedestrian (negative). From this positive/negative classification, we extracted the following information:

- True Positives (TP) are the images labeled as positive and classified as positive, i.e., the image contains a person, and the classifier result is pedestrian.
- True Negatives (TN) are images labeled as negative and classified as negative, i.e., the image does not contain a person, and the classifier result is a non-pedestrian.
- False Positives (FP) are labeled as negative and classified as positive, i.e., the image does not contain a person, but the classifier result is a pedestrian.
- False Negatives (FN) are sample images labeled as positive and classified as negative, i.e., the image contains a person, but the classifier result is non-pedestrian.

Such information allows the extraction of some metrics that provide a fair comparison between the classifiers. The accuracy [46] is a measure of the correct classifications on the test set samples. In other words, accuracy indicates how many correct answers are provided by the classifiers considering all classes. In the target application, a higher rate of correct answers is very important because this enables a more precise search and rescue planning, which may lead to an increasing number of rescues and also a lower operational cost. Such a metric is a good measure when the dataset is balanced, i.e., the number of samples of all classes is the same or there are a small difference [46]. The accuracy is expressed by the following equation:

(3) $$Accuracy=\frac{TP+TN}{TP+TN+FP+FN}$$

The sensitivity (or recall) [46] measures the proportion of positive samples classified as positive. In other words, sensitivity indicates how many correct answers are provided by the classifiers, but only considers the positive samples. In the target application, sensitivity indicates whether the classifier is able to detect the higher number of pedestrians correctly. A higher rate indicates that, when the classifier detects a pedestrian, the search and rescue team can safely send resources to that location in order to rescue the person since the probability of success is high. The sensitivity is expressed by the following equation:

(4) $$Sensitivity=\frac{TP}{TP+FN}$$

On the other hand, specificity [46] measures the proportion of negative samples classified as negative. In other words, specificity indicates how many correct answers are provided by the classifiers, but only considering the negative samples. In the target application, specificity indicates that the classifier is able to classify a higher number of non-pedestrian images correctly. A higher rate indicates that, when the classifier does not detect a pedestrian, the search and rescue team can ignore that location. Thus, this saves time and resources, since there is a low probability of finding a person in that location. The specificity is expressed by the following equation:

(5) $$Specificity=\frac{TN}{TN+FP}$$

Considering the requirements of search and rescue missions, it is worth highlighting that sensitivity and specificity metrics are more suitable for classification performance assessment rather than using the precision metric [46]. In a search and rescue mission, it is more important not to miss a person than to classify the images into the two classes correctly.

#### 5.1.2. Results

We evaluated the classifiers by executing them on the test set using a batch process. The batch file contains a list of files that were used in the experiment and the respective class labels. Thus, it is possible to compare the classifier output with the real image classification. Table 3 shows the results.

As the test set contains balanced classes, the accuracy analysis shows the real classifiers’ performance. Using the accuracy as the main metric, the CNN2 showed better results, which were close to 100%, while LBP cascade achieved just 66.15%. We improved the results in comparison to our previous work [4], in which we used a smaller number of images to train the CNN. Actually, CNN1 achieved a result similar to [4], presenting a difference of only 0.01%. This low difference shows that CNN1 is effective at classifying people in aerial images, as much as CNN2, even though the CNN1 architecture extracts a lesser number of features. When the models present similar results, the simple one is the best solution to the problem [44]. Such a simpler model is important for another reason: computing time performance. CNN1 presents a better computational performance because it has a smaller number of layers and neurons (see Section 4.4). Moreover, CNN1 has a smaller number of convolutional filters, which also impacts the computing time performance. However, CNN2 tends to achieve a better generalization, since it extracts more data from the image, due to the number of convolutional layers.

HOG + SVM also achieved good results, as well, which might be an indication of the reasons for it being widespread in people classification applications [10,34,57,58,59]. Despite the wide variety of poses with various rotation, translation and scale situations, the SVM was effective and achieved a high generalization rate. The sensitivity and specificity rates show HOG + SVM as a conservative classifier, presenting a high TP rate and a low FN rate. The results are similar to other studies [10,34] that focus on pedestrian detection in aerial images, where the rates are close to 90%. Another advantage of SVM is the low training and classification computing time (see Section 5.4). CNN outperformed SVM, because of its capacity to automatically define and extract features. Unlike HOG, which is a descriptor of contour, CNN is able to define different sorts of features based on textures, colors, regions, contour, and so on.

In comparison with CNN and SVM, the two cascade implementations achieved unsatisfactory results. The accuracy with LBP and Haar features was close to 70%. The specificity shows low rate results, mostly in LBP cascade with a 54.8% rate. These results indicate a liberal classifier, i.e., to achieve high TP rates, the classifier has a high FP rate. The low efficacy of these classifiers is related to the texture characteristics. In problems such as pedestrian detection in aerial images, the objects of interest (pedestrians) present few pixels. Since LBP and Haar are defined as texture descriptors, a low number of pixels in the textures cannot define representative features for the classifiers. Thus, such a characteristic strongly influences the classification capacity. Furthermore, the samples used for training and testing present a wide variety of textures, affecting the generalization of the cascade classifier.

### 5.2. Experiment 2: Occlusion Experiments

#### 5.2.1. Overview

**Figure 11:**
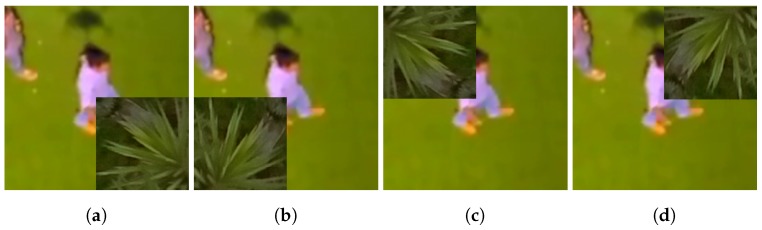
Samples of occlusion experiments: (**a**) occlusion on bottom-right quadrant; (**b**) occlusion on bottom-left quadrant; (**c**) occlusion on top-left quadrant; (**d**) occlusion on top-right quadrant.

In order to perform a more comprehensive evaluation, we executed some experiments to evaluate the classifiers in situations of partial occlusion, i.e., an object of the scene covers up the pedestrian. For this experiment, we selected 8067 pedestrian samples from the GMVRT-v1 and GMVRT-v2 datasets, including samples from both the training and validation sets. We simulated the occlusion using a very common element in aerial images: a tree. The tree image covers up 25% of the pedestrian, i.e., the tree image is 1/4 of the sample size. Therefore, the pedestrian samples were divided into four equal parts (i.e., four quadrants), and thus, the occlusion element (i.e., the tree image) was inserted once in one of these parts. Thus, this process generates four new test samples from one original sample. As we used only positive samples in these experiments, the evaluation was carried out by comparing the accuracy and sensitivity metrics (see Section 5.1.1). Figure 11 depicts some images used as test samples in the occlusion experiments.

#### 5.2.2. Results

Similar to the classification experiments, the occlusion experiments followed the same protocol: a batch file with the file list and the label were processed. As we defined four occlusion regions (top right, top left, bottom right and bottom left), these experiments evaluate the model generalization and the classifier classification performance when part of the pedestrian image is removed. It is worth considering that the occlusion situations are common in images obtained by aerial platforms.

In these experiments, we used only positive images (pedestrian images), and thus, the accuracy metric is the most representative. As we have only TP and FN, the accuracy and sensitivity values are similar. To achieve a fair comparison, we executed the classification procedure on the original images (without occlusion). Table 4 shows the classification results for each occlusion region, whereas Table 5 shows the results for the original images classification and also the metrics’ average considering the four occlusion regions.

As one can see, CNN2 achieved a consistent performance. CNN2 results show that such an implementation provides a more robust classifier for all situations, since the accuracy rate is similar in all occlusion situations, with an average of 70%. On average, the CNN2 results outperform CNN1 by more than 30%, showing the efficiency of a higher number of features. These features are the number of convolutional layers and the filter sizes inside the layers of CNN2. Moreover, CNN2 has filters that extract more local features in comparison to the number of global features of CNN1. These local features are not an advantage in all sorts of object recognition applications. The local features are indicated for applications in which small visual details about the object are required [22], which seems to be the case for pedestrian detection in aerial images. Nonetheless, it is important to notice that a CNN with local features has more features, resulting in an increasing number of parameters to be adjusted in network training, and thus, it increases the effort in configuring such a classifier.

Excluding CNN2, the other classifiers achieved an unsatisfactory performance, below (or very close to) 50%. As the problem has only two classes, these classifiers can be considered worse than a random classifier, which has 50% of a chance of providing the correct classification. The main problem in CNN1 is the feature extraction process, which is not enough to generalize the problem when some parts of the object are occluded. With HOG + SVM, we achieved 50% accuracy, with instability in different occlusion regions. The HOG features use the gradient orientation of the edges in images; thus, occluding some part of the image strongly impacts the accuracy rate. The cascade classifiers presented poorer classification results in occlusion situations because the algorithm has problems identifying the objects with a reduced number of pixels. Indeed, the problem is even worse with occlusion regions since the number of pixels of the target objects decreases drastically.

### 5.3. Experiment 3: Classification Performance of Pattern Recognition System Implementations

#### 5.3.1. Overview

As mentioned, we performed two distinct evaluations. Firstly, we assessed the classification performance of the PRS implementations (see Section 4.5). The goal is to assess the complete PRS workflow of these implementations. For that, we used the metrics presented in Section 5.1. This assessment has been divided into two parts: segmentation techniques and the ten PRS implementations.

#### 5.3.2. Results

In the first experiment, we evaluated the performance of the segmentation and detection algorithms: Saliency Map (SM) and Thermal Image Processing (TIP). This experiment evaluates the number of ROI identified by each algorithm before the classification step.

The classification is the most time-consuming step of the PRS, and hence, it is important to avoid the execution of unnecessary classifications. Therefore, it is important to select only the ROI that present a higher probability of depicting the target object (in this work, pedestrians) and discard the other ROI.

**Figure 12:**
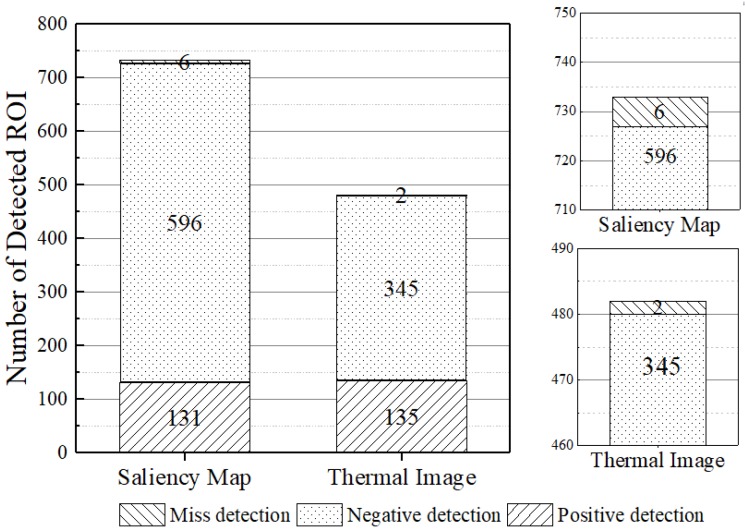
Number of positive, negative and missed detections for each segmentation algorithm.

The segmentation algorithm must detect the maximum number of target objects. In search and rescue missions, the pedestrian detection is critical; a faster detection may be the difference between saving or losing a life. To perform this experiment, we captured a total of 137 scenes, in which a person appears in distinct environments. For that, we flew a quad-rotor UAV carrying one Raspicam and one FLIR thermal camera; we captured 137 images from each camera. Figure 12 shows the results of segmentation techniques executed on these RGB and thermal images.

**Figure 13:**
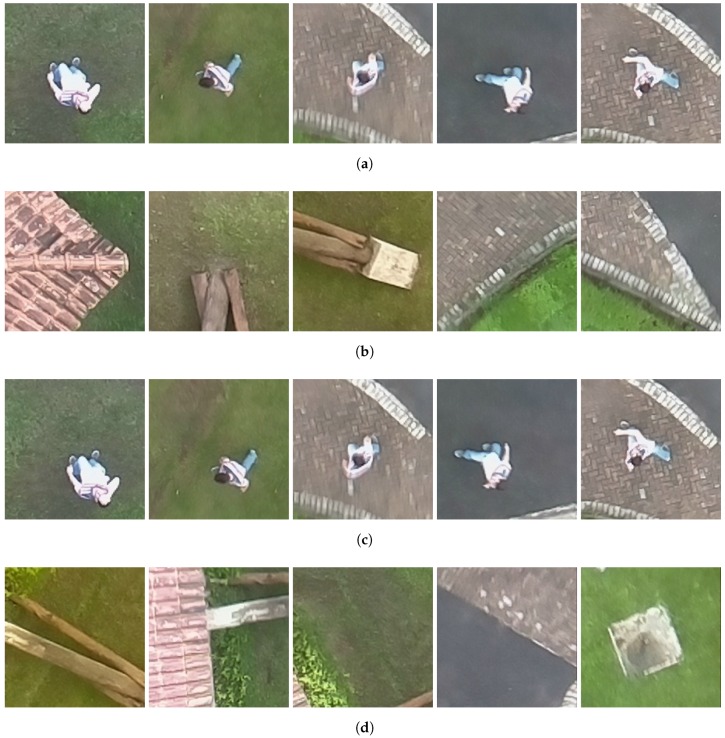
Samples of ROI: (**a**) positive and (**b**) negative samples extracted with saliency map; (**c**) positive and (**d**) negative extracted with thermal image processing.

One can see that both techniques detect ROI that contain non-target objects, i.e., non-pedestrians ROI. In spite of that, this result is better than the one achieved with traditional techniques such as sliding window [40,59]. The results show that SM detects more ROI in comparison with the TIP. Indeed, TIP finds 42% less ROI; these ROIs contain non-target objects. Thus, the PRS with SM as the segmentation and detection algorithm requires more time for processing since it has more ROI to classify, even though the ROI do not present any target object. This conclusion is confirmed in the computing time performance experiment (see Section 5.4). Moreover, by using SM, a PRS has a higher probability of generating incorrect classifications, since more ROI will be submitted to the classification step. As one can see, both techniques present a low number of misses to detect the ROI containing the target objects (i.e., pedestrians). This result is also better than traditional techniques. Figure 13 shows some examples of ROI extracted using the segmentation algorithms.

In the second experiment, we assessed the classification performance of the PRS implementations as a whole. The results are presented in Table 6, which shows the number of true positives, true negatives, false positives, false negatives, as well as the sensitivity and specificity metrics.

The PRS classification results are similar to the ones obtained in the classifier performance experiment (see Section 5.1). The PRS implementations that use TIP (in the segmentation and detection step) show lower sensitivity due to a small translation caused by the segmentation process. The translation is the reason for the resolution difference between the thermal and RGB images. In order to extract the ROI in the RGB images, Equations (1) and (2) (see Section 4.5) are used to calculate the centroid coordinates of the RGB ROI based on the centroid coordinates of the thermal images’ ROI. As the resulting ROI centroid coordinates are not integer values, these rational values are rounded up to the next integer value, and thus, the difference in the decimal values creates the small translation variation.

Considering the specificity results depicted in Table 6, SM indeed increases the probability of classification error (i.e., specificity is lower in all PRS that use SM) since the number of FP and TN is higher in comparison with PRS that use TIP. Comparing these results with the ones obtained in the detection experiments (see Section 5.1.2), the two CNN implementations, Haar cascade, and LBP cascade, achieved comparable results. However, only the CNN kept the results close to a 90% rate. On the other hand, one can see a decrease in the classification performance of HOG + SVM: almost 20% percentage points less in both sensitivity and specificity. HOG + SVM performance decreases due to the shape variations of the ROI extraction. The HOG features do not perform well when there are high variations in object shape [57]. When the thermal image is used, the translation effect produced during the ROI extraction in the RGB image negatively influences the classifier performance.

Analyzing the classifiers results, we confirm that CNN performs a better classification. Regardless of the translation variations of TIP, the classifier is not affected, keeping the lower error rates since the sensitivity and specificity are close to 90%. Despite the high sensitivity, the Haar cascade and LBP cascade maintained unsatisfactory performance, showing an expressive number of false positives. HOG + SVM presented a significant difference in performance by using SM and TIP due to the translation effects.

### 5.4. Experiment 4: Computing the Time Performance of Pattern Recognition System Implementations

#### 5.4.1. Overview

Applications such as search and rescue missions or video surveillance present real-time constraints (it is important to mention that we used the term “real-time” to indicate predictable execution time rather than a fast (or instantaneous) execution time in the average case, but without a bounded execution time in the worst case executing time [60]). Providing information in real-time allows a fast decision-making process to move rescue teams and other resources since the PRS response time is bound and known a priori. In this sort of application, the data collected by the UAV can be processed in two ways: (i) an embedded system sends the processed information to the rescue team leader or (ii) a Mobile Ground Control Station (MGSCS) (e.g., a portable computer) receives the images captured by the UAV and processes them.

Processing the images on the embedded system has some advantages especially in the data transmission. If the data are processed onboard, the UAV sends only the geographic information to the rescue team, decreasing the communication bandwidth demand. However, if the embedded system does not have enough computational resources (e.g., processing capacity and memory) to provide the information processed in real-time, the information sent by the UAV might be useless for the rescue teams since the decision-making process can be compromised due to late or delayed information [61]. The MGCS usually has more computational resources to process the data (e.g., a faster CPU, more memory or even an integrated GPU). However, it requires better communication infrastructure to receive the images captured by the UAV. A poor communication link may take too much time to transmit the images from the UAV to the MGCS, or the data integrity or security could be compromised, leading to noisy images or even fake images.

These experiments focus on evaluating the computing time performance of the ten PRS implementations. The goal is to assess how long these implementations take to provide the results. It is important to evaluate whether the implementations execute within an acceptable time on a low-cost computing system, so it is possible to decrease the overall component cost of the PRS. Therefore, we have defined some premises: (i) the communication between the UAV and MGCS is in real-time [60]; (ii) the communication link provides enough bandwidth; and (iii) the data are transmitted and received in a consistent way without any noise.

We executed the ten implementations on two different hardware platforms: an inexpensive single-board embedded computer (Raspberry PI 2) and a Mobile Ground Control Station (MGCS). The Raspberry Pi 2 is an inexpensive, yet capable embedded computer that supports multiple peripherals such as cameras and other sensors. The quad-core ARM Cortex-A7 processor with 900 MHz and 1 GB of RAM allows efficient parallel implementations. The Raspberry Pi 2 simulates the execution of the PRS onboard the UAV. We choose this board due to its popularity and low cost. On the other hand, for the MGCS, we used an Intel Core i5-3210M 2.5-GHz laptop with 4 GB RAM and Linux Ubuntu 14.04 64 bit, but no GPU. We used OpenBLAS, an open-source implementation of Basic Linear Algebra Subprograms (BLAS) [62], in both platforms. The BLAS is a specification that establishes a set of low-level routines to execute linear algebra operations, like matrix operations, scalar product, linear combinations and matrix product. The OpenBLAS is optimized for several architectures, including ARM and x86, and supports multi-threading implementations. Thus, OpenBLAS can be seen as a suitable tool for multi-core systems such as the ones used in these experiments.

In order to measure the execution time for each PRS step, we used the C++ function *GetTickCount*() available in OpenCV library. This function presciently returns the current number of clock cycles. The computing time of a code snippet can be measured by calling *GetTickCount()* twice: before the first statement and after the last statement. Then, we calculated the duration of code snippet execution in clock cycles and converted it to milliseconds. It is worth highlighting that each segmentation algorithm can find various ROI candidates. Therefore, the segmentation algorithm’s computing time is calculated as the average of the computing time of each identified ROI. To minimize the influence of operating system interference, we executed each experiment 20 times and calculated the average computing time.

We defined the computing time performance as one frame per second (fps), feasible to detect a pedestrian in aerial imagery in a search and rescue mission supported by UAV. Naturally, the greater the fps, the better, but a PRS implementation must reach a minimum of 1 fps to be considered suitable for use in this kind of application.

**Figure 14:**
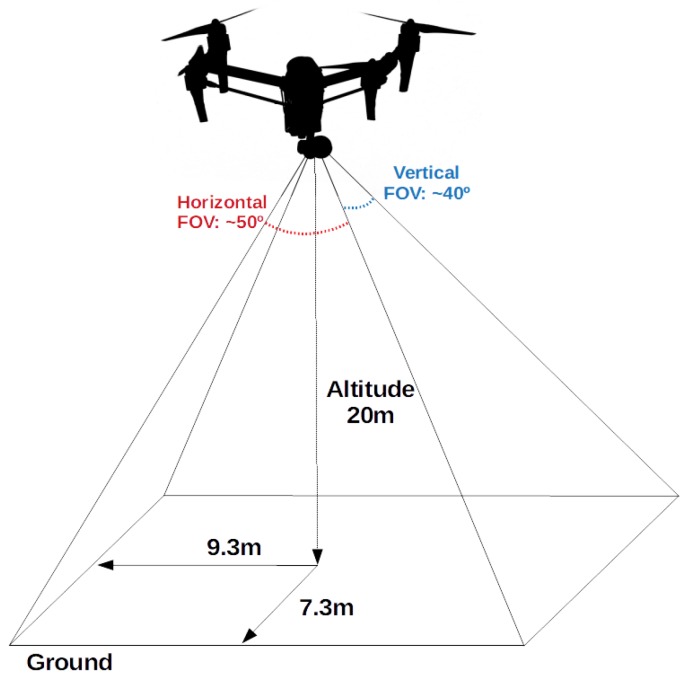
The UAV field of view at 20 m of altitude.

Such a soft real-time constraint is based on the angle of the cameras Field Of View (FOV) (the cameras’ FOV are defined as horizontal and vertical angles), the flight altitude and speed. The thermal camera specifications indicate 51${}^{\circ }$ as the horizontal angle and 40${}^{\circ }$ as the vertical angle, whereas the Raspicam specification indicates 53.5${}^{\circ }$ and 41.41${}^{\circ }$, respectively. The experiments were conducted at 20–25 m of altitude with a quad-rotor UAV with 14 m/s as the maximum speed (this is the average speed of a number of quad-rotor UAV that is available for purchase). It is important to mention that flying at higher altitudes would allow covering a greater terrain area. However, onboard cameras will capture fewer details from the terrain, including a reduced number of pixels per pedestrian, which may reduce the PRS efficacy as demonstrated in the occlusion experiments (see Section 5.2.2). Therefore, by using the triangle rectangle properties, it is possible to determine the terrain area that was captured during the experiments, i.e., we calculate the ground FOV. Figure 14 shows the FOV, angle relations and flight altitude used in these experiments. For the envisaged worst case scenario, in which the UAV flies at 20 m of altitude, the ground FOV is a rectangle of approximately 18.6 m (horizontal) by 14.6 m (vertical). Thus, considering a maximum speed of 14 m/s, no visual information is lost in any direction of the UAV movement. Actually, in this case for vertical FOV, the direction is an overlap of 0.8 m and 4.8 m for horizontal FOV direction. However, it is important to highlight that for other sorts of pedestrian detection applications, e.g., autonomous car driving or surveillance/tracking, 1 fps is an insufficient performance, since the dynamics of these applications demand a shorter response time. Since the UAV itself does not need this information (i.e., pedestrian detection) to perform any maneuver, detecting pedestrians at 1 fps seems sufficient because the system can send the GPS location of the person within a low latency for the search and rescue team, which, in turn, will plan and act accordingly within a scale of minutes.

#### 5.4.2. Results

Table 7 shows the computing time performance of the PRS implementations executed on the embedded platform, i.e., on the Raspberry Pi 2. In summary, the PRS executing on the embedded system cannot achieve 1 fps; the exception is the PRS implemented HOG + SVM and the TIP that reached 14 fps in the worst case scenario. Despite the use of techniques to reduce the number of executions of the classification step, the Raspberry Pi 2 does not have the processing capacity necessary to process the majority of the PRS implementations at 1 fps. In the previous experiments, HOG + SVM does not achieve the best classification results, especially in occlusion experiments. Nonetheless, HOG + SVM + TIP provides a solution with a suitable classification performance in the situation without occlusions and good computing time performance. When the probability of occluded objects is high, HOG + SVM may not be a good solution, although it achieved such a good computing time performance. It is important to notice that the HOG + SVM seems a good option when the low-cost hardware is required since it achieved 14 fps in Raspberry Pi 2 (Table 7). Additionally, in comparison to CNN, the SVM has much fewer parameters, wherein a linear kernel depends only on the number of features. In our experiments, while the SVM defined 8100 kernels (the number of HOG features), CNN2 had 61 million parameters. This difference (in the number of parameters) impacts the computational performance because SVM executes much fewer algebraic operations. Furthermore, to the best of our knowledge, neither OpenCV nor OpenBLAS implementations use the Raspberry PIś GPU. Hence, this performance result might be better when such a GPU-supported implementation will be released.

Table 8 shows the computing time performance of the PRS implementations executing on the MGCS, i.e., the Intel i5-based laptop without GPU. These results show that some PRS reached more than 1 fps. In most cases, the speedup was more than 10 times in comparison with the embedded system results. Since the classifiers had more computational resources available in MGCS, the time to process the images decreased. The TIP outperforms the SM in regard to the computing time. The SM in the worst-case scenarios takes 250 ms to process each image of 1280 × 720 pixels. As one can see, in several PRS implementations, 250 ms is more than 25% of the entire system computing time; in the PRS implemented with HOG + SVM, this represents 75% of the whole computing time. On the other hand, TIP does not present a significant time impact since it represents only 1% of the whole computing time. However, it is important to highlight that a significant disadvantage of TIP using the low-resolution thermal camera is the environment temperature. When the environment temperature is greater than 30 Celsius degrees, which is very common in tropical regions, it is not possible to identify the persons in the image since there is a small difference between the human body and the environmental temperatures, i.e., there is a small difference in the pixel values. In this sense, SM presents fewer restrictions since it uses the RGB images whose pixels are related to the objects’ light emission. In spite of that, one must be aware that SM has problems identifying the saliency when the object has similar colors. Despite the high time for processing, SM seems better indicated, as it can be used in more situations (including warm weather). Therefore, SM allows the PRS to achieve 1 fps or more even in a low-cost computing platform.

Considering the computing time of the entire PRS, one can see some interesting results. The computing time experiments showed that it is possible to use CNN without using GPU (but using a CPU with some degree of parallelism) while achieving a frame rate suitable for detecting pedestrians in aerial images for search and rescue applications. The experiments show an evident difference in the CNN architectures and implementations. The number of layers and feature filters directly influence the processing time. Despite the 1 fps achieved in the PRS implemented with CNN2 and TIP, the results are close to the 1-fps constraint.

On the other hand, PRS implemented with HOG + SVM is able to process the images at 100 fps in the worst case scenario; this can be considered a very good performance result, as already discussed. The PRS implemented with cascade classifiers shows quite good performance results, achieving at least 3 fps. However, as mentioned, computing time performance is not the only factor to consider. Classification performance is probably the most important factor. In general, the results show something close to the “no free lunch theorem” [63], i.e., engineers need to perform a trade-off analysis to choose a balance between a better classification or a shorter computing time. In this scenario, HOG + SVM emerges as the suitable candidate to be used in a low-cost computing platform, since it has a reasonable classification rate with faster time processing in spite of its poor performance when there is occlusion of the target objects.

## 6. Conclusions

New applications that employ UAV have emerged due to the popularity and cost reduction of UAV platforms, especially multi-rotor UAV. One of these applications is the detection of pedestrians in aerial imagery using computer vision and pattern recognition techniques. This application can be used in many situations such as search and rescue missions and surveillance. Search and rescue missions require much time and effort from the rescue teams, especially when the analysis of the captured image is done manually by a human being. Such a repetitive and error-prone task can be executed autonomously by a computer vision system. In this work, we developed and evaluated various pattern recognition systems (based on [44]) for an autonomous pedestrian detection system. These PRS have a sequence of steps that include: image acquisition, detection and segmentation, feature extraction, classification and post-processing. We implemented each step with different techniques. For the segmentation and detection algorithms, we used: saliency map and thermal image processing. In feature extraction and classification, we used: HOG + SVM, LBP cascade, Haar cascade and two CNN architectures. We performed several experiments to assess the implemented PRS in terms of their classification capacity and their respective computing time performance. We evaluated both the entire PRS implementations and also some of their steps separately. The goal was to assess the feasibility and suitability of executing these PRS on low-cost computing systems such as embedded systems or regular computers without a dedicated GPU.

The experiments reveal exciting results, especially in classification performance assessment. By using the CNN, we achieved the best classification rates, which were close to 100%. This seems to justify the increasing interest in using CNN in computer vision applications. The results obtained in the HOG + SVM experiments show that such an approach is suitable and robust for people classification tasks. HOG + SVM was able to classify with more than a 90% accuracy rate in spite of the presence of variations in the perspective and object poses and shapes. On the other hand, the cascade classifiers presented the worst results: less than a 70% accuracy rate. This classification performance seems to indicate that texture-based feature descriptors are not suitable for pedestrian detection in aerial images. In the experiments that included object occlusion, CNN2 showed impressive results, achieving more than 70% accuracy. This accuracy indicates good performance in spite of the occlusion of 25% on the ROI that depicted the pedestrians. The others classifier achieved only 50% or less in their classification rate in the occlusion situations, indicating a classification performance closer to a random classification.

The combination of computer vision techniques used to implement different PRS has also been assessed in terms of classification capacity and computing time performance. We assessed the entire PRS and also some of its steps. We performed some experiments with segmentation and detection techniques, namely SM and TIP. Both techniques show advantages and disadvantages in several aspects. SM detects more ROI, which presents a direct impact on the number of objects to be classified, demanding more computational resources to process all selected information. On the other hand, SM presents a better precision of the segmented ROI as an advantage, since this leads to less translation, improving some classifiers’ results such as HOG + SVM. TIP requires fewer computing resources as it detects a lower number of ROI, and hence, it submits less ROI to the classification step, decreasing the computing time as a whole. However, TIP presented some issues during image segmentation related to some translation effects that influence the classification step. In addition, TIP has some limitations associated with the environmental conditions. For instance, in environments with a temperature greater than 30 Celsius degrees, the thermal camera used is not able to detect the thermal signature of a human being. Finally, the detection and segmentation techniques influence the PRS performance since they reduce the number of objects submitted to classifiers. Other popular approaches as sliding windows require that several ROI be classified, which consumes much time and computational resources. Using the sliding window with ROI of 256 × 256 and with a one-pixel step in images of 1280 × 720, it would be necessary to classify 476,625 ROI. Using the HOG + SVM to classify all ROI of the sliding window in images of 1280 × 720 would require more than 330 s.

We have also assessed the execution time of the PRS on two platforms: an embedded system (a Raspberry Pi 2) and a Mobile Ground Control Station (a notebook with Intel Core i5-3210 2.5 GHz without a dedicated GPU). In general, the experiments carried out with the embedded system did not show good results because only the PRS implemented with HOG + SVM + TIP achieved a performance of at least 1 fps. On the other hand, most PRS implementations executed on the MGCS achieved a performance of more than 1 fps. Segmentation and detection steps have a considerable influence on the PRS computing time performance. SM is responsible for a great percentage of the total PRS computing time, whereas TIP used only 1% of the total execution time. Since the classification is the most time-consuming step in PRS, the detection algorithm influences the entire system performance: the lower the number of segmented ROI, the shorter the time (as a whole) needed to classify them. In the classification step, PRS implemented with CNN presented a higher execution time (but the best classification performance), followed by the cascades and HOG + SVM, respectively. It is important to highlight that HOG + SVM+TIP achieved an impressive result of 100 fps when executed on the MGCS and 14 fps on the embedded system both without using GPU acceleration. However, it is important to highlight that HOG + SVM classification performance degrades when there is occlusion of the pedestrian.

As future work, we will continue studying CNN, since it has shown great potential for pedestrian detection, even in situations with partial occlusion. Despite demanding more computational resources, our CNN implementation can be optimized and used in real-world applications by means of using GPUs, FPGAs or even special resources of ARM processors to improve parallelism. The NVIDIA Jetson platform seems to be a good option to use in future works since it provides a GPU embedded into a small-sized printed circuit board. Such a parallel hardware can provide the required computing time performance for the CNN-based PRS for pedestrian detection in aerial imagery. Another option is the optimization of CNN architectures by using techniques to reduce the number of parameters and multiplications with floating-point operations.

## Figures and Tables

**Table 1:** CNN1 architecture details.

	Conv1	Pool1	Conv2	Pool2	Conv3	Pool3
Input map	3	32	32	64	64	128
Input	227 × 227	110 × 110	55 × 55	49 × 49	24 × 24	18 × 18
Output map	32	32	64	64	128	128
Output	110 × 110	55 × 55	49 × 49	24 × 24	18 × 18	9 × 9
Filters	10 × 10	2 × 2	7 × 7	2 × 2	7 × 7	2 × 2
Step	2 × 2	2 × 2	1 × 1	2 × 2	1 × 1	2 × 2

**Table 2:** CNN2 architecture details.

	Conv1	Pool1	Conv2	Pool2	Conv3	Conv4	Conv5	Pool3
Input Map	3	96	96	256	256	384	384	256
Input	227 × 227	55 × 55	27 × 27	27 × 27	13 × 13	13 × 13	13 × 13	13 × 13
Output Map	96	96	256	256	384	384	256	256
Output	55 × 55	27 × 27	27 × 27	13 × 13	13 × 13	13 × 13	13 × 13	6 × 6
Filters	11 × 11	3 × 3	5 × 5	3 × 3	3 × 3	3 × 3	3 × 3	3 × 3
Stride	4 × 4	2 × 2	1 × 1	2 × 2	1 × 1	1 × 1	1 × 1	2 × 2
Zero Padding	0	0	2	0	1	1	1	0

**Table 3:** Classifier evaluation results.

Classifier	# TP	# TN	# FP	# FN	Accuracy	Sensitivity	Specificity
CNN1	2245	2219	29	3	99.29%	99.87%	98.70%
CNN2	2248	2235	13	0	99.71%	100%	99.42%
HOG + SVM	2068	2086	162	180	92.36%	91.99%	92.73%
Haar Cascade	1705	1604	644	543	73.48%	75.85%	71.10%
LBP Cascade	1740	1241	1007	508	66.15%	77.40%	54.80%

**Table 4:** Experiment results grouped by occlusion regions.

	Top Right			Top Left			Bottom Right			Bottom Left		
**Classifier**	**# TP**	**# FN**	**Acc**	**# TP**	**# FN**	**Acc**	**# TP**	**# FN**	**Acc**	**# TP**	**# FN**	**Acc**
CNN1	4001	4066	49.6%	4150	3917	51.4%	2467	5600	30.6%	1993	6074	24.7%
CNN2	5767	2300	71.5%	6239	1828	77.3%	5168	2899	64.1%	5763	2304	71.4%
HOG + SVM	6006	2061	74.5%	4701	3366	58.3%	2129	5938	26.4%	3517	4550	43.6%
Haar Cascade	1949	6118	24.2%	2221	5846	27.5%	2093	5974	25.9%	2093	5974	25.9%
LBP Cascade	2203	5864	27.3%	2307	5760	28.6%	2186	5881	27.1%	2249	5818	27.9%

**Table 5:** Classification performance on the original images versus the occluded images.

	Original Images			Occlusion Average		
	**# TP**	**# FN**	**Acc**	**# TP**	**# FN**	**Acc**
CNN1	8055	12	99.9%	3152.75	4914.25	39.1%
CNN2	8067	0	100.0%	5734.25	2332.75	71.1%
HOG + SVM	7511	556	93.1%	4088.25	3978.75	50.7%
Haar Cascade	6104	1963	75.7%	2089	5978	25.9%
LBP Cascade	6731	1336	83.4%	2236.25	5830.75	27.7%

**Table 6:** Classification performance of all PRS implementations.

		# TP	# TN	# FP	# FN	Sensitivity	Specificity
CNN1	Saliency Map	121	530	66	10	92.37%	88.93%
	Thermal Image	119	344	2	16	88.15%	99.42%
CNN2	Saliency Map	112	545	51	19	85.50%	91.44%
	Thermal Image	103	344	1	32	76.30%	99.71%
HOG + SVM	Saliency Map	97	476	120	34	74.05%	79.87%
	Thermal Image	85	302	43	50	62.96%	87.54%
Haar Cascade	Saliency Map	81	393	203	50	61.83%	65.94%
	Thermal Image	75	262	83	60	55.56%	75.94%
LBP Cascade	Saliency Map	99	363	233	32	75.57%	60.91%
	Thermal Image	92	248	97	43	68.15%	71.88%

**Table 7:** Time estimation of PRS implementation in the Raspberry Pi 2 platform.

	Segmentation		Classification		Total		
	**Average (s)**	**Max (s)**	**Average (s)**	**Max (s)**	**Average (s)**	**Max (s)**	**fps**
SM + CNN1	3.67±0.57	4.21	0.73±0.1	0.79	8.81±1.32	11.12	0.09
TIP+ CNN1	$8\times 10^{-3}\;\pm \;3\times 10^{-5}$	$7\times 10^{-4}$	0.77±0.01	0.81	2.75±0.82	5.34	0.18
SM + CNN2	3.69±0.5	4.25	2.1±0.2	2.1	9.8±2.3	13.23	0.08
TIP + CNN2	$7\times 10^{-3}\;\pm \;4\times 10^{-5}$	$7\times 10^{-4}$	2.02±0.4	2.38	6.08±2.28	11.86	0.08
SM + HOG	3.65±0.61	4.14	$1\times 10^{-2}\;\pm \;2\times 10^{-4}$	0.01	3.85±0.02	3.87	0.25
TIP + HOG	$8\times 10^{-3}\;\pm \;3\times 10^{-5}$	$7\times 10^{-4}$	$1\times 10^{-2}\;\pm \;8\times 10^{-3}$	0.01	0.04±0.02	0.07	14
SM + Haar	3.66±0.63	4.16	0.38±0.07	1.01	6.45±0.76	9.37	0.11
TIP + Haar	$8\times 10^{-3}\;\pm \;3\times 10^{-5}$	$7\times 10^{-4}$	0.39±0.08	0.59	3.7±0.8	7.97	0.13
SM + LBP	3.73±0.4	4.07	0.4±0.09	1.1	6.5±0.9	9.69	0.11
TIP + LBP	$8\times 10^{-3}\;\pm \;5\times 10^{-5}$	$7\times 10^{-4}$	0.42±0.07	0.53	3.81±0.77	7.08	0.14

**Table 8:** Time estimation of PRS implementation in the MGCS platform.

	Segmentation		Classification		Total		
	**Average (s)**	**Max (s)**	**Average (s)**	**Max (s)**	**Average (s)**	**Max (s)**	**fps**
SM + CNN1	0.23±0.01	0.27	0.08$\;\pm \;2\times 10^{-3}$	0.08	0.77±0.13	0.74	1.35
TIP + CNN1	$6\times 10^{-5}\;\pm \;3\times 10^{-5}$	$6\times 10^{-5}$	0.08$\;\pm \;1^{-3}$	0	0.22±0.08	0.41	2.43
SM + CNN2	0.23±0.03	0.22	0.18$\;\pm \;4\times 10^{-3}$	0	1.63±0.32	1.4	0.71
TIP + CNN2	$4\times 10^{-4}\;\pm \;3\times 10^{-5}$	$6\times 10^{-5}$	0.17±0.01	0	0.61±0.19	0.93	1.08
SM + HOG	0.21±0.04	0.23	$7\times 10^{-4}\;\pm \;10^{-4}$	$9\times 10^{-4}$	0.28±0.02	0.3	3.33
TIP + HOG	$7\times 10^{-5}\;\pm \;3\times 10^{-5}$	$6\times 10^{-5}$	$7\times 10^{-4}\;\pm \;10^{-4}$	$9\times 10^{-4}$	0.02$\;\pm \;1\times 10^{-3}$	0.02	100
SM + Haar	0.22±0.01	0.22	0.03±0.01	0.08	0.42±0.06	0.61	1.64
TIP + Haar	$8\times 10^{-5}\;\pm \;3\times 10^{-5}$	$6\times 10^{-5}$	0.04±0.01	0.06	0.13±0.05	0.28	3.57
SM + LBP	0.22$\;\pm \;3\times 10^{-3}$	0.26	0.04±0.02	0.11	0.51±0.08	0.76	1.31
TIP + LBP	$8\times 10^{-5}\;\pm \;5\times 10^{-5}$	$6\times 10^{-5}$	0.04$\;\pm \;4\times 10^{-3}$	0.8	0.14±0.04	0.26	3.85

## Abbreviations

The following abbreviations are used in this manuscript:

CNN	Convolutional Neural Network
FN	False Negative
FP	False Positive
fps	Frames per second
FOV	Field of View
HOG	Histogram of Oriented Gradient
GPU	Graphics Processing Unit
LBP	Linear Binary Patterns
MBD	Movement-Based detection
MGCS	Mobile Ground Control Station
PRS	Pattern Recognition System
ROI	Region Of Interest
SBD	Stationary-Based Detection
SM	Saliency Map
SVM	Support Vector Machine
TIP	Thermal Image Processing
TN	True Negative
TP	True Positive
UAV	Unmanned Aerial Vehicle
